# Identification and sequence analyses of the gliding machinery proteins from *Mycoplasma mobile*

**DOI:** 10.1038/s41598-020-60535-z

**Published:** 2020-03-02

**Authors:** Isil Tulum, Kenta Kimura, Makoto Miyata

**Affiliations:** Department of Biology, Graduate School of Science, Osaka City University, Sumiyoshi-ku, Osaka, 558-8585 Japan

**Keywords:** Cell biology, Microbiology

## Abstract

*Mycoplasma mobile*, a fish pathogen, exhibits its own specialized gliding motility on host cells based on ATP hydrolysis. The special protein machinery enabling this motility is composed of surface and internal protein complexes. Four proteins, MMOBs 1630, 1660, 1670, and 4860 constitute the internal complex, including paralogs of F-type ATPase/synthase α and β subunits. In the present study, the cellular localisation for the candidate gliding machinery proteins, MMOBs 1620, 1640, 1650, and 5430 was investigated by using a total internal reflection fluorescence microscopy system after tagging these proteins with the enhanced yellow fluorescent protein (EYFP). The *M. mobile* strain expressing a fusion protein MMOB1620-EYFP exhibited reduced cell-binding activity and a strain expressing MMOB1640 fused with EYFP exhibited increased gliding speed, showing the involvement of these proteins in the gliding mechanism. Based on the genomic sequences, we analysed the sequence conservativity in the proteins of the internal and the surface complexes from four gliding mycoplasma species. The proteins in the internal complex were more conserved compared to the surface complex, suggesting that the surface complex undergoes modifications depending on the host. The analyses suggested that the internal gliding complex was highly conserved probably due to its role in the motility mechanism.

## Introduction

Class *Mollicutes* is mainly represented by *Mycoplasma* species which are parasitic or occasionally commensal bacteria that have small cell size, small genomes, and no peptidoglycan layer^1,2^. More than ten *Mycoplasma* species show gliding motility. Interestingly, mycoplasma gliding was not connected to flagella, pili, or other bacterial motility systems. This might be due to the loss of the peptidoglycan layer during mycoplasma evolution, because the conventional bacterial motility systems need to be anchored to the peptidoglycan layer^3^. Moreover, the motility combined with the ability to adhere to the host cell surface play a key role in its pathogenesis^4^.

**Figure 1:**
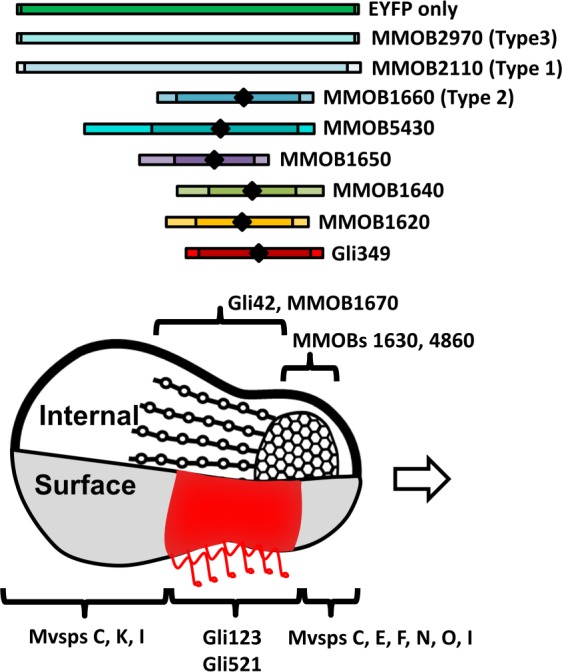
Schematic illustration of *M. mobile* cell with distribution of fluorescence signals. The surface and internal structures are illustrated in a cell schematic with protein localisation. The distribution of fluorescence signals obtained in this study are summarized as coloured boxes in the upper side with the positions of peak (diamond) and half (vertical line) signal intensity, for proteins indicated in the right. Gli349 and other proteins were detected by an antibody and EYFP fusion, respectively.

**Figure 2:**
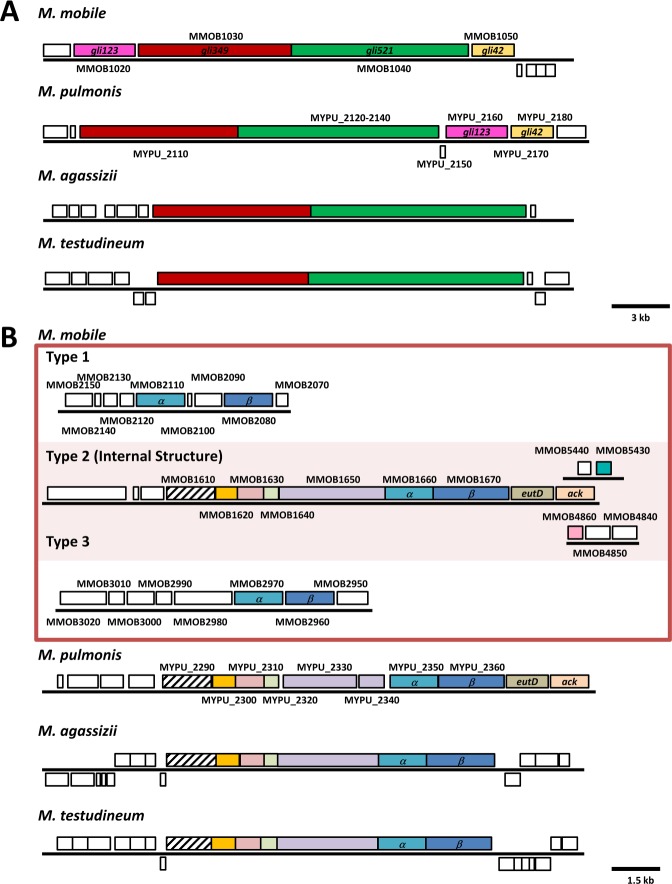
Comparison of genes involved in the gliding motility of *M. mobile* with other genes in the *Hominis* group. Homologous genes are indicated by boxes of the same colour. Gene names and locus tags are indicated inside and outside of the boxes, respectively. The schematic diagrams were generated by using Molligen and JGI databases. (**A**) Analyses of genes coding surface structures. *gli123* and *gli42* genes are coded in a region neighbouring to *gli349* and *gli521* in the genomes of *M. mobile* and *M. pulmonis*, whereas their orthologs were not found on the genomes of *M. agassizii* and *M. testudineum*. (**B**) Analyses of genes coding internal structures. Three types of gene clusters related to F-type ATPase/synthase in *M. mobile* are shown in the red box. Type 2 codes for the internal structure of the gliding machinery. Gene clusters for three species homologous to the internal structure of *M. mobile* gliding machinery are shown in the lower positions. All of these species have five (six in case of *M. pulmonis*, because the gene homologous to MMOB1650 is split into two ORFs) conserved hypothetical proteins coded from the upstream of the genes of α- and β- subunit paralogs. The phosphate acetyltransferase (*eutD*) and acetate kinase (*ack*) genes are predicted to be transcribed as an operon with the α- and β- subunit paralogs in *M. mobile and M. pulmonis*^15,43^, while they are distantly positioned in the genomes of *M. agassizii*, and *M. testudineum*.

*Mycoplasma mobile*, a fast-gliding mycoplasma was isolated from the gills of freshwater fish (Fig. 1). It exhibits gliding motility with an average speed of 2.0 to 4.5 μm/s^5–7^. *M. mobile* gliding machinery can be divided into two parts, internal and surface structures^5,8^. The surface structure is composed of three huge proteins, Gli123, Gli349, and Gli521 (123, 349, and 521 kDa molecular weights, respectively). Gli42 (42 kDa) should be translated together with the surface proteins but the localisation on the surface has not been clarified. These proteins are involved in the gliding machinery (Fig. 2A)^9–13^. Previous studies identified, MMOBs 1620, 1630, 1640, 1650, 1660, 1670, 0150, 4530, 4860, and 5430 as the components of the internal structure by mass spectrometry (Fig. 2B)^8,14^. The involvement of MMOBs 1630, 1670, and 4860 in the gliding machinery was shown by immunofluorescence microscopy and the involvement of MMOB 1660 was shown by EYFP tagging^9^. Seven of these proteins were tandemly coded in a locus, in the order of MMOBs 1610, 1620, 1630, 1640, 1650, 1660, and 1670 from the 5′ end. MMOBs 1660 and 1670 are paralogs of the α and β subunits of F-type ATPase/synthase, respectively. Although, the other five proteins, MMOBs 1610, 1620, 1630, 1640, and 1650 did not show obvious sequence similarity with other components of F-type ATPase/synthase, the secondary structure prediction and the three-dimensional modeling of the structures suggested similarities between MMOB1630 and MMOB2090, the γ subunit^5,9^. The a and c subunits of F-type ATPase/synthase were characterised by the frequent appearance of the transmembrane segments, while the b and δ subunits consisted of transmembrane segments and coiled-coil parts, suggesting that the MMOBs 1610 and 1620 were derived through gene fusion, respectively from the ‘a and c’ and ‘b and δ’ subunits of F-type ATPase/synthase.

MMOBs 0150 and 4530 were assigned as the xylose solute binding protein and phosphoglycerate kinase, respectively. MMOBs 4860 and 5430 had no annotations^8,14^.

F-type ATPase/synthases have been identified in most bacteria, including *Mycoplasma*. *Mollicutes* genomes that have been examined to date, other than those of ureaplasmas and phytoplasmas, contain a typical, complete operon encoding the subunits of the F-type ATPase/synthase^15^. In *Mycoplasma*, this genuine F-type ATPase/synthase referred to as “Type 1” is responsible for membrane potential maintenance based on ATP hydrolysis^15^. Interestingly, in addition to the genuine F-type ATPase/synthase (Type 1), F-type ATPase/synthase subunit homologs have been identified in mycoplasma genomes flanked with characteristic neighbouring genes. Phylogenomic studies classified these homologs into two types of F-type ATPase/synthase clusters, namely Type 2 and Type 3^15^. Type 2 cluster was found only in two related species from the *Hominis* group: *M. mobile* and *Mycoplasma pulmonis*, the rodent pathogen. Interestingly, three of the seven proteins (eight in case of *M. pulmonis*) of the *M. mobile* Type 2 F-type ATPase/synthase are involved in the internal structure of the gliding machinery.

In the present study, the subcellular localisation of component candidates, MMOBs 1620, 1640, 1650, and 5430 was examined by fluorescent protein tagging using total internal reflection fluorescence (TIRF) microscopy^16^. We found that the fusion protein expression influenced the binding activity and gliding speed for MMOBs 1620 and 1640, respectively. Thus, to summarise we concluded the involvement of at least eight proteins in the internal structure involved in gliding and analysed their amino acid sequences.

## Results

### Subcellular localisation of the internal structure proteins

**Figure 3:**
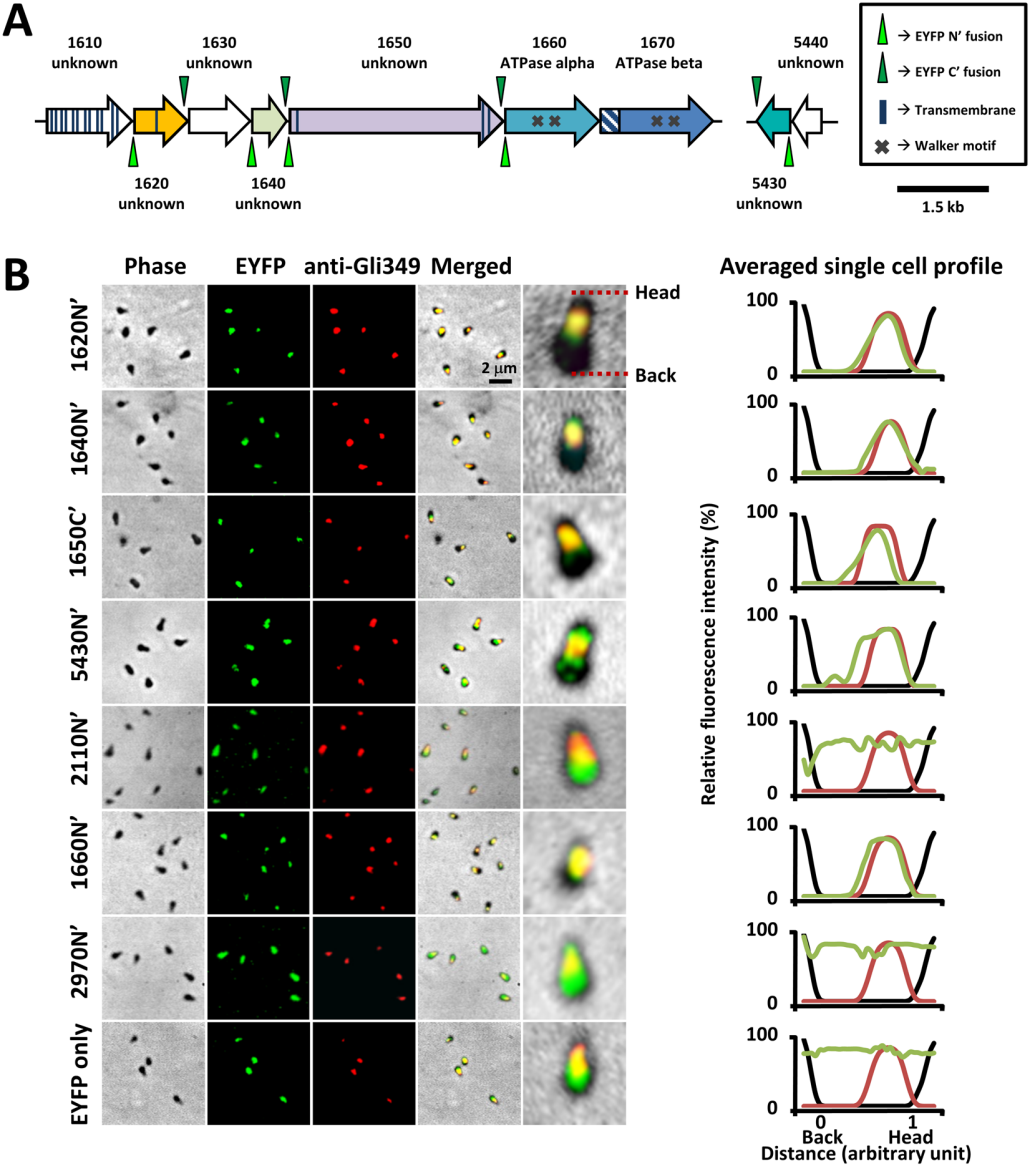
Subcellular localisation of the candidate proteins of the internal structure. (**A**) Schematic of gene organisation and fusion proteins. MMOB locus tags and gene annotation are shown for each gene. The positions of the fluorescent tagging for the generation of fusion proteins are indicated by triangles. (**B**) Fluorescence image and profile for MMOBs 1620, 1640, 1650, 5430, 2110, 1660, 2970, and EYFP only. The images of phase-contrast microscopy, EYFP fluorescence, and Gli349 immunofluorescence are shown in the three left panels. Merged images along with the magnified images are shown in the right two panels. Representative profile for an average of 10 cell images is shown in the right panel. The densities in the phase-contrast image, the fluorescence of EYFP fusion, and the fluorescence of Gli349 are indicated by black, green and red lines, respectively.

In order to examine the involvement of candidate proteins in the gliding machinery, we tagged MMOBs 1620, 1640, 1650, and 5430 with a yellow fluorescent protein. Previously, we had optimised and developed an EYFP tagging system for *M. mobile*^9,17,18^. The system included an *eyfp* gene which was codon-optimised for *Mollicutes* (*opteyfp*). To achieve higher fluorescence intensity, *opteyfp* was placed under the control of a strong promoter of the gene encoding for the elongation factor Tu of *M. mobile* (*Mtuf*) resulting in *Mtuf-opteyfp* fragment. In the present study, the *Mtuf-opteyfp* fragment was inserted into pMTnGm, a Tn4001 transposon-derived vector system that harbours transposase (*tnp*) gene outside of the transposable element by using the Infusion subcloning method. The resulting fluorescence may be dependent on whether the tag was fused at N or C- terminal of the protein. Considering this possibility, we created N-terminally and C-terminally tagged fusion proteins as different clones (Fig. 3A).

The C-terminal fusions of MMOBs 1620, 1640, and 5430 and the N-terminal fusion of MMOB1650 showed weak signals which are not suitable for analyses (Fig. S1). Then, the N-terminal fusions of MMOBs 1620, 1640, 5430 and the C-terminal fusion of MMOB 1650 were selected for further analyses. Next, we examined the subcellular localisation of candidate proteins, MMOBs 1620, 1640, 1650, and 5430 by comparing their positions relative to Gli349, which is known to localise at the gliding machinery (Fig. 3B)^12,13^. Gli349 was labelled by Cy3 using a monoclonal antibody, mAb7^12,13^.

**Figure 4:**
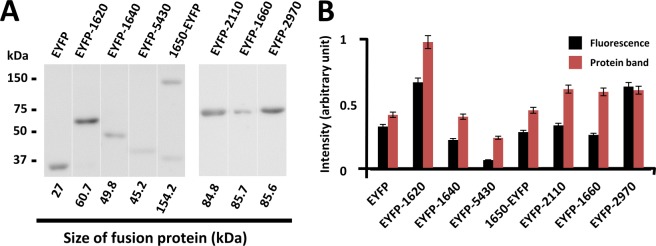
Comparison of fluorescence and expression level of EYFP protein. (**A**) Detection of EYFP fusion protein by immunoblotting. The molecular sizes are shown on the left. The calculated molecular sizes are shown below the band images. (**B**) Comparison of protein band intensity and fluorescence. An equal amount of sample (obtained from an equal number of cells) was loaded in each lane. The values were averaged for three independent experiments and shown with standard deviations (SDs).

For localisation controls, we examined the localisation of Type 1 and Type 3 F-type ATPase/synthases, the α-subunit paralogs. MMOB2110 (Type 1) and MMOB2970 (Type 3) were tagged at their N-terminus by EYFP, as previously done for MMOB1660 (Type 2)^9^. Next, we confirmed the expression of the fusion proteins by immunoblotting using an anti-EYFP antibody. We detected EYFP-MMOB1620, EYFP-MMOB1640, MMOB1650-EYFP, EYFP-MMOB5430, EYFP-MMOB2110, EYFP-MMOB1660, and EYFP-MMOB2970 (Fig. 4A) at their expected molecular size. MMOB1650-EYFP exhibited two bands, one of them was the expected size, while the other band was of smaller molecular size (Fig. 4A), suggesting that MMOB1650-EYFP was either processed or digested in the cell. The amounts of EYFP and fusion proteins were consistent with their fluorescence intensities, suggesting that the fluorescence intensities of directly reflected the expression levels of the fusion proteins (Fig. 4B).

Further, we estimated the fluorescence intensity of a cell with a typical flask shape in phase-contrast along the cell axis and determined the distribution range (Fig. S2)^17^. To map the fluorescent foci on the cell axis, the phase-contrast image was used to determine the front-end position of a cell assuming that the internal structure was uniform in shape and density among individual cells. The fluorescence intensities were measured for 10 images of each construct and the average intensity was calculated (Fig. 3B). The signal of MMOB1660 overlapped with that of Gli349, as reported previously^9^. The signals of MMOBs 1620, 1640, and 1650 mostly overlapped with that of Gli349. The signal of MMOB5430 also overlapped with Gli349, but extended beyond Gli349 in the direction of the cell body. The signals of the α-subunit paralogs, MMOBs 2110 and 2970, were distributed through the cell, distinct from those of MMOB1660 and other component candidates.

### Effects of fusion proteins on cell binding and gliding speed

**Figure 5:**
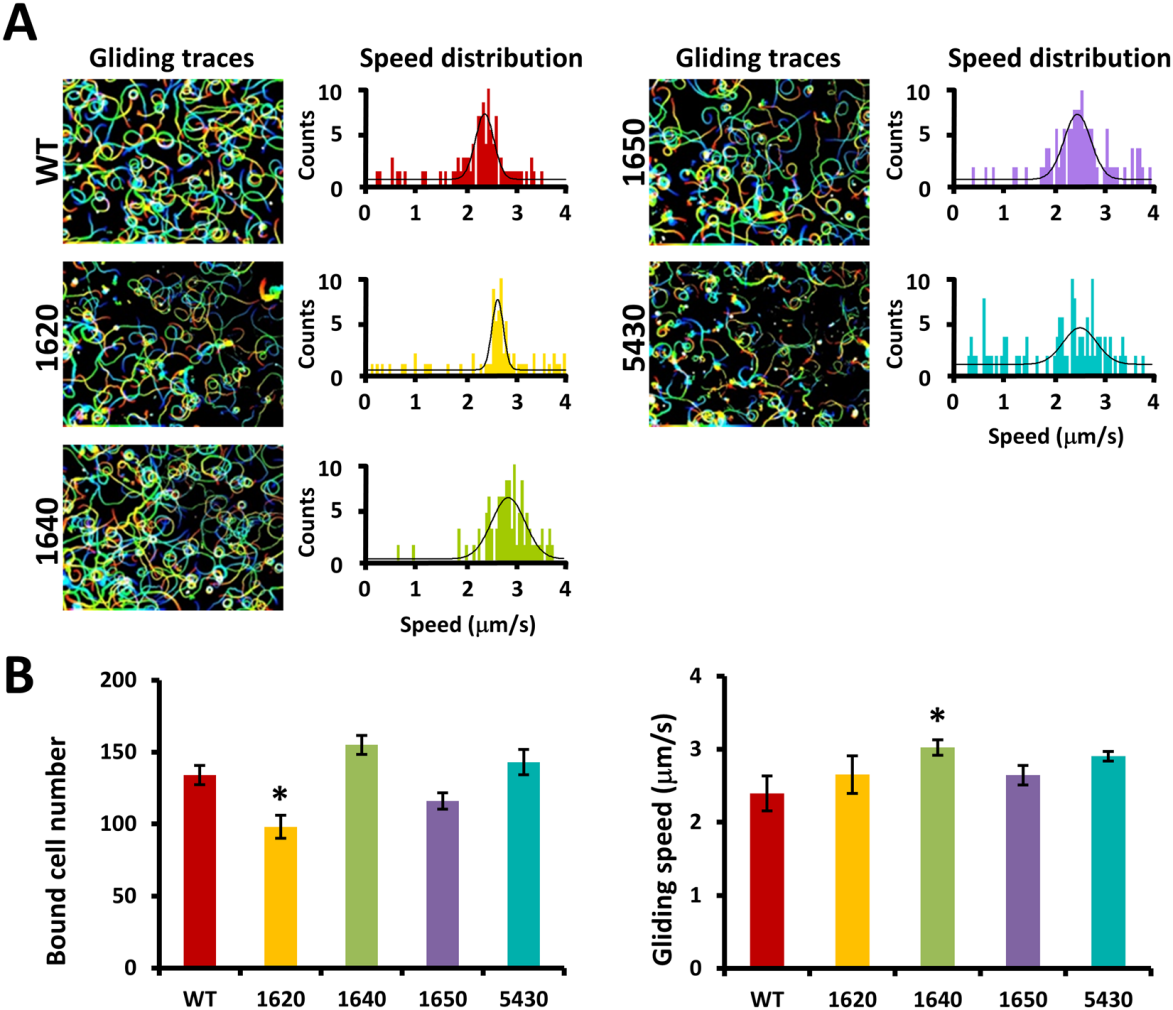
Gliding and binding properties of strains expressing the fusion proteins of MMOBs 1620, 1640, 1650, and 5430. (**A**) Cell trajectories are presented as a stack for 10 s, with colour variation from red to blue. Distribution of gliding speeds averaged for 30 s at 1-s intervals was fitted with a Gaussian curve (n = 120). (**B**) Ratios of cell binding and gliding speed in various strains. Averages of cell-binding activity (left) and gliding speed (right) with SDs are shown. The SD was judged by Student’s *t*-test (p < 0.05) and marked with an asterisk (*) for values significantly different from the WT.

The fluorescent protein tag on the fusion protein might cause steric hindrance in the target proteins affecting their function^9,17,18^. To examine the effects of fluorescence tagging for the candidate proteins, the gliding motility, the binding activity and the gliding speed of transformant cells were examined and compared to the wild-type (WT) strain. All strains were cultured to an optical density of 0.08 at 600 nm. Then, cells were collected, washed, and suspended in phosphate-buffered saline supplemented with glucose (PBS/G). The cell suspensions were inserted into tunnel slides, and videos were recorded on a phase-contrast microscope (Fig. 5A). An area of glass 64.0 μm wide and 85.3 μm long was selected and the cells bound to it were counted. The cells from 10 independent fields were counted for each strain. The average gliding speed was calculated for 30 s and 120 independent cells including fusions of MMOBs 1620, 1640, 1650, and 5430. The cells with MMOB1620-EYFP exhibited a 25% reduced binding activity and the cells with EYPF-MMOB1640 exhibited 33% increased gliding speed (Fig. 5B). These results are consistent with MMOBs 1620 and 1640 being a part of the gliding machinery, with changes in their functions caused by the fluorescent protein tagging, affecting some steps in the gliding mechanism.

### Genome and sequence analyses of the new gliding species

The class *Mollicutes* represented by *Mycoplasma* are subdivided into four subgroups. Some species in the *Pneumoniae* and *Hominis* subgroups have gliding capability^1^. These two subgroups are phylogenetically distant from each other and do not share any genes involved in gliding^5,6,19–21^. The orthologs of *M. mobile* gliding proteins have been reported in the genome of *M. pulmonis*, but not in other species^10^. To obtain information related to protein functions based on the amino acid sequences, we conducted a bioinformatic search for the candidate protein orthologs in the genomes of other mycoplasmas. First, we searched for the orthologs of Type 2 F-type ATPase/synthase α subunit of *M. mobile* using BLASTP (Accelerated protein-protein BLAST) and PSI-BLAST (Position-Specific Iterated Blast) algorithms within *Mycoplasma* (taxid:2085) non-redundant protein sequence database. We could not find any other Type 2 F-type ATPase/synthase α-subunit orthologs. Then, we searched for the draft genome sequences in the Joint Genome Institute genome portal and Type 2 F-type ATPase/synthase gene clusters were identified along with Type 1 and Type 3 clusters in *Mycoplasma agassizii* and *Mycoplasma testudineum* (pathogens of the desert tortoise), which belong to the *Hominis* subgroup (Fig. 2A).

Further, we compared the Type 2 gene clusters of *M. mobile, M. pulmonis, M. agassizii*, and *M. testudineum*. All of these species except *M. pulmonis* have five conserved hypothetical proteins upstream from the α- and β- subunit paralogs. The ortholog of MMOB1650 is split into two genes in *M. pulmonis*, MYPU_2330 and MYPU_2340. Two genes, phosphate acetyltransferase (*eutD*) and an acetate kinase (*ack*) are predicted to be transcribed as an operon with the α- and β- subunit paralogs in *M. mobile and M. pulmonis*, but these genes were distantly positioned in the genomes of *M. agassizii* and *M. testudineum*.

Next, we searched for the genes coding for the surface proteins of gliding machinery in the genomes of *M. agassizii*, and *M. testudineum* (Fig. 2B). In the genome of *M. mobile*, the genes for the surface structure are present in the order of *gli123-gli349-gli521-gli42*. This gene alignment is mostly conserved in *M. pulmonis*. We analysed *M. agassizii* and *M. testudineum* genomes by BLAST search and found only the *gli349-gli521* genes, but not the *gli123* and *gli42* genes were not detected in the genome of *M. agassizii* and *M. testudineum*.

### Sequence analyses of the surface structure proteins

**Figure 6:**
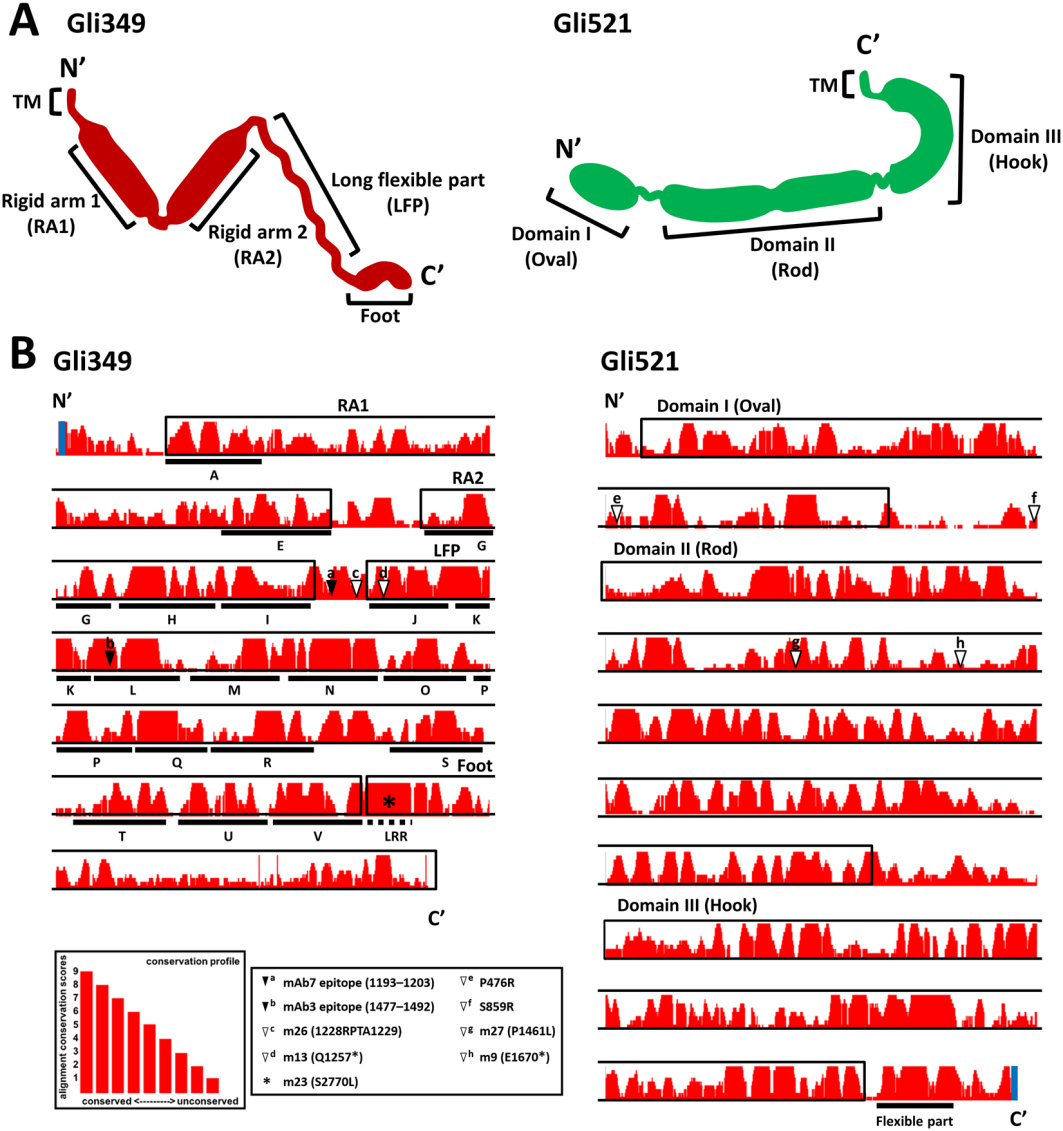
Characteristics of surface gliding proteins, Gli349 and Gli521. (**A**) Schematic diagrams of the Gli349 and Gli521 molecules. Gli349 consists of the Transmembrane segment (TM), Rigid arm 1 (RA1), Rigid arm 2 (RA2), Long flexible part (LFP) and Foot from the N-terminus. Gli521 consists of four parts, Domains I (Oval), II (Rod), and III (Hook) and TM. (**B**) Characterisation of sequences. TMs are indicated by blue bars. The region corresponding to each part of the schematic is indicated by box and name for RA1, RA2, LFP, Foot, Domain I (Oval), Domain II (Rod), and Domain III (Hook). Repeat sequences and a leucine-rich repeat of Gli349 are indicated by bars marked A through V and a dashed line marks LRR, respectively. The degree of conservation of the amino acid sequence in each column defined in T-Coffee is indicated by the length of red bars shown in the left lower side. The height of the column presents the variation according to the score: fully conserved (9), unconserved (1). The working points of the antibodies inhibiting gliding motility are indicated by black triangles, shown as a and b for the epitopes of mAb7 and mAb3, respectively. Mutations affecting gliding motility are marked by white triangles, shown as c: m26 (1228RPTA1229) in Gli349; d: m13 (Q1257*) in Gli349; e: P476R in Gli521; f: S859R in Gli521; g: m27 (P1461L) in Gli521; and h: m9 (E1670*) in Gli521. The mutation S2770 (m23) in Gli349, essential for the binding activity, is indicated by an asterisk (*).

By using the multiple sequence alignment algorithm T-Coffee, we compared the amino acid sequences of Gli349 and Gli521 orthologs from *M. mobile*, *M. pulmonis*, *M. agassizii*, and *M. testudineum* genomes (Fig. 6) to examine the relationship of the orthologs and to predict their function.

Gli349 protein (3183 amino acids) of *M. mobile* functions as a “leg” during gliding and is essential for binding and gliding^12,13,22,23^. The Gli349 protein is composed of two short rigid arms, a long flexible part, and a C-terminal oval ‘foot’ which are linked tandemly (Fig. 6A)^24–26^. A foldable hinge links the two short arms. An additional feature of Gli349 is that it has 18 repeats of about 100 amino acids^26^.

The sequence alignment of the four Gli349 orthologs showed 72 similarity in the main score of T-Coffee. The N-terminal region, Rigid arm 1 (RA1) comprising the repeat sequences A through E (118–727 aa) showed 62 T-Coffee score. Rigid arm 2 (RA 2) comprising the repeat sequences G through I (830–1161 aa) showed a 74. The long flexible part (LFP) comprising the repeat sequences J through V (1248–2720 aa) showed 80 T-Coffee score. The binding positions of the previously described two inhibitory antibodies mAb3 and mAb7, and two mutation points inhibiting binding and gliding were found on Gli349 in m13 and m23 strains^23,27^. These constituted the highly conservative regions corresponding to J-K-L (1248–1546 aa) repeats in the LFP. The N-terminus of the foot has a leucine-rich repeat (LRR), as observed in Toll-like receptors and contains a serine residue (at position 2770) which is essential for binding^5,22^. The regions corresponding to J-K-L showed a 95 T-Coffee score (Fig. 6B)^23,27^.

Gli521 protein (4727 amino acids) of *M. mobile*, is also essential for binding and gliding, functions as a crank in force transmission through interaction with Gli349 (Fig. 6A)^11,28,29^. Limited proteolysis showed that Gli521 consists of three parts, domain I (oval, 44–715 aa), domain II (rod, 889–3035 aa) and domain III (hook, 3244–4560 aa) which are connected by flexible hinges^28^. The full-length protein sequence alignment comparing Gli521, WP_084232748, WP_094254639, and MYPU_2120–2140 showed 65 T-Coffee score, while domain I, domain II, and domain III showed 72, 77, and 77, respectively. The gap regions between the domains showed low similarities (~ 41 T-Coffee score). The flexible region at the C-terminal end of domain III corresponding to 4573–4662 amino acids showed high similarity (70 T-Coffee score), indicating that this flexibility was essential for the protein function. Previously, our group has identified mutations and epitopes in the monoclonal antibody against Gli521 which influenced binding activity and gliding speed. Interestingly, these sequences were never mapped onto the highly conserved regions^23,27^. We then focused on analysing the conservativity of individual mutation points to understand its effect on the function of the protein. Proline at 476^th^ and serine at 859^th^ positions in Gli521 are totally diverse but proline at 1461^st^ position is totally conserved among the four species. Serine at 2770^th^ position in Gli349 is totally conserved, consistent with the fact that m23 mutant which has this serine mutated to leucine is incapable of binding^23,27^. Using genome sequences of two new species, we evaluated the conservativity of each position. This will give us a clue to design new experiments to elucidate this unique gliding mechanism.

### Phylogenetic relationship analysis of the gliding proteins

**Figure 7:**
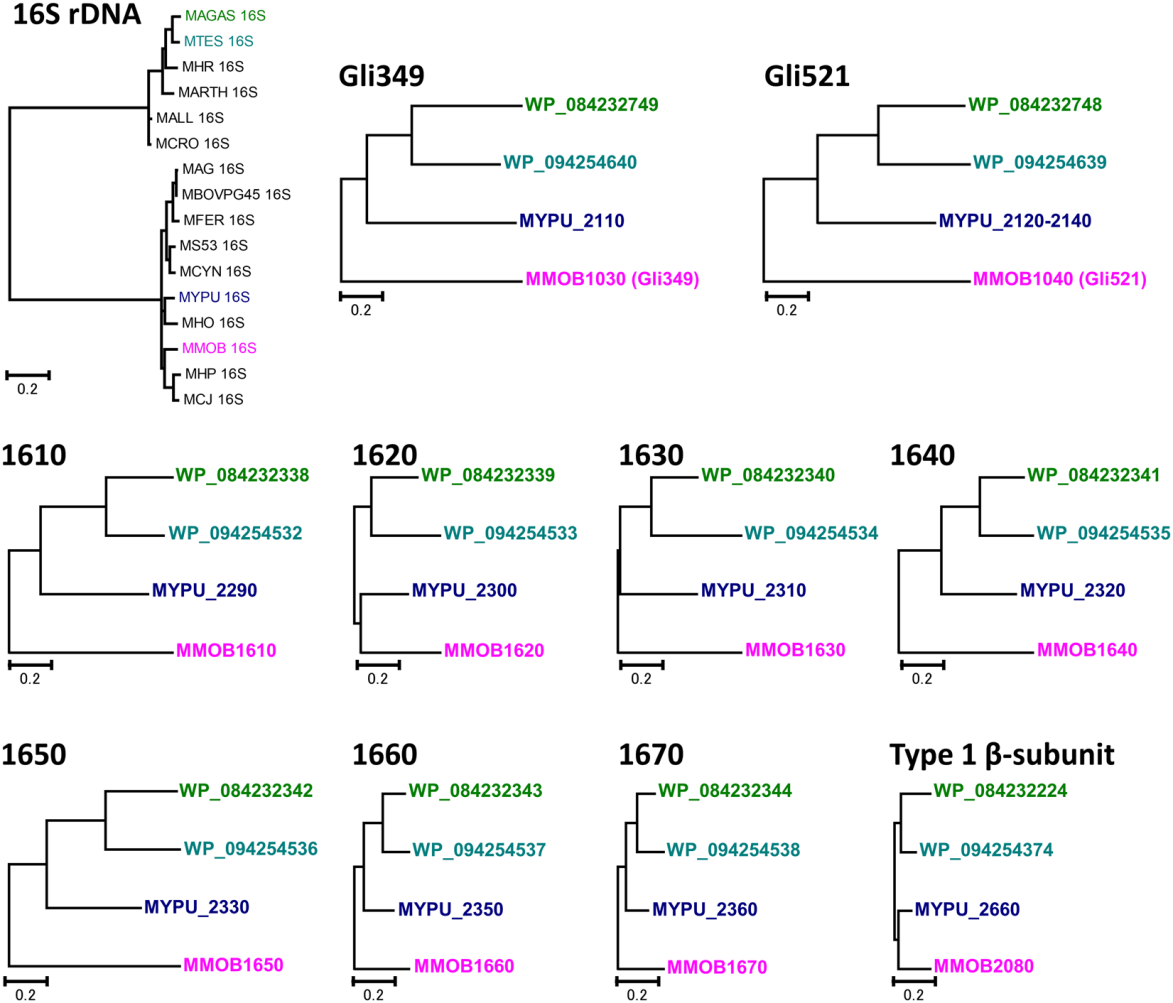
Phylogenetic trees of the protein components in gliding machinery and 16S rDNA in the *Hominis* group. Phylogenetic trees were generated by the ML method with the “gap complete deletion” option chosen by using the MEGA7 package. The trees were drawn to scale, with branch lengths measured for the number of substitutions per site. The genes are shown by their locus tags and colour-coded as follows: *M. mobile*: magenta, *M. pulmonis*: dark blue, *M. testudineum*: light blue, *M. agassizii*: green. See Table S1 for details.

The phylogenetic trees of Gli349 and Gli521 and the proteins of the internal structure were generated using the maximum likelihood (ML) method (Fig. 7)^14,30^. The topologies of the phylogenetic trees were identical for orthologs of Gli349, Gli521, MMOBs 1610, 1630, 1640, 1650, 1660, and 1670 orthologs and common with that of tree based on the 16S rDNA sequences. The orthologous proteins of *M. testudineum* and *M. agassizii* were positioned on sister branches, showing the closest relationship between these species. These two organisms were more closely related to *M. pulmonis* than *M. mobile*. The relationships among individual protein groups of *M. mobile*, *M. pulmonis*, *M. agassizii*, and *M. testudineum* were supported by 100% probability as per the bootstrap analysis. We were unable to find any orthologs of Gli123 and Gli42 in *M. testudineum* and *M. agassizii*, and orthologs of MMOBs 5430 and 4860 in other species except in *M. mobile*. Our observations suggested the existence of a conserved core overlaid with species-specific diversity.

## Discussion

The protein components in the internal structure of gliding machinery were analysed. The MMOB5430 signal was slightly different from other component proteins, as shown in Fig. 3B. This difference in the signal pattern might be due to the small size of MMOB5430. In general, compared to the larger proteins smaller proteins tend to occupy smaller spaces in the cell, EYFP tagging to such proteins might result in steric clashes with other components leading to the formation of aggregates from the excess protein molecules and ultimately resulting in patchy signals as seen for MMOB5430^9,17,18^. However, cellular localisation based on the fluorescent signals showed the involvement of MMOB5430 in the gliding machinery.

The EYFP fusions caused a reduction in cell binding for MMOB1620 and acceleration in gliding speed for MMOB1640. These results can be explained if we consider the working model explaining the gliding mechanism, where a cycle consists of four steps of interaction including catch, pull, drag and release^5,6,8,27,31^ with the binding target, i.e. sialylated oligosaccharides (SOs)^32–34^. The EYFP fusion to MMOB1620 might reduce the catch step or activate the release step, and the EYFP fusion to MMOB1640 may activate the frequency of the pull step.

We have shown that 10 proteins localised to the gliding machinery of *M. mobile*. Proteins MMOBs 1620, 1630, 1640, 1650, 1660, 1670, 5430, and 4860 constitute the internal structure, and Gli123 (MMOB1020), Gli349 (MMOB1030), and Gli521 (MMOB1040) constitute the surface structure. Another component protein of the gliding machinery, Gli42 (MMOB1050) is translated together with the surface proteins, but the localisation has not been clarified. Although we do not have any direct evidence for MMOB1610, we think that it is probably involved in the gliding machinery, because proteins transcribed as an operon frequently form a complex in bacteria. It is likely that the internal structure of gliding machinery evolved as Type 2 F-type ATPase/synthase gene cluster from the genuine cluster^5,8,15,22^. As structural details of all the internal complex are not available, it is difficult to establish the similarities with F-type ATPase/synthase but the rough image obtained by electron microscopy suggested a pair of particles featured by rotational symmetry^8,14^ which can be related to the feature of F-type ATPase/synthase^15,35^.

Further, we performed sequence analyses of gliding proteins based on an amino acid sequence from the four species. We acquired the genomic sequence of two new *Mycoplasma* species, which glide probably by a mechanism common with *M. mobile*. We were able to investigate conservativity in each region of the proteins for the first time by comparing the amino acid sequences of the proteins required for gliding from four *Mycoplasma* species (Figs. 2, 6, 7). The summary of the analysis is as follows: (i) The proteins of internal structure are highly conserved compared to the proteins of the surface structure. The surface structure should change depending on the environments, as observed in the difference in the conservativity levels between the surface and core structures of bacterial flagellins^36^. (ii) Gli123 and Gli42 were not essential for the mechanism of gliding, while Gli349 and Gli521 were essential. (iii) The N-terminal region in the foot domain of the Gli349 protein was well conserved, suggesting that it should be essential for gliding^5,22^. (iv) The phylogenetic relationship of each protein is in common with the phylogenetic relationship of 16S rDNA, suggesting that the gliding mechanism evolved with the *Mycoplasma* genomes.

In this study, we identified new proteins involved in the internal and surface machineries for an *M. mobile* gliding mechanism. This information will be a clue to clarify the mechanism of this specialized gliding mechanism.

## Materials and Methods

### Strains and culture conditions

*M*. *mobile* 163 K (ATCC 43663) strain was cultured in Aluotto medium at 25 °C^37,38^. For the selection of *M*. *mobile* transformants, gentamicin sulfate was used at a final concentration of 50 μg/ml^9,37–39^. *Escherichia coli* strain DH5α and Stellar cells were used for all DNA manipulation procedures^9^.

### Plasmid construction and transformation

For pMTnPE construction, *tuf-eyfp* was amplified from pMobopt using primers: 5′-CACACGAATTCTTAAAAAAGCTTGAACATAAGA-3′ and 5′-CACACGTCGACGAGCTCGGCCTATATGGCCAGATCTTTTATATAATTCATCCATACCTAAT-3′. The amplified *tuf-eyfp* fragment was inserted into pMTnGm using *Eco*RI and *Sal*I sites. *M. mobile* genomic DNA was prepared using the Genomic-tip System (Qiagen, Hilden, Germany). For the construction of the N-terminal fusions of each targeted gene (MMOBs1620, 1640, 1650, and 5430), the DNA fragments were amplified from the genomic DNA and inserted into the *Bgl*II-*Sac*I sites (the 3′ end of *eyfp* gene) of pMTnPE. For the construction of the C-terminal fusions of each targeted gene, the amplified DNA fragments were inserted into the 5′ end of the *eyfp* gene by using In-Fusion EcoDry PCR Cloning Kit (TaKaRa Bio, Shiga, Japan). Electroporation, colony PCR, and transposon insertion site detection were performed as previously described^9,17,18,40^.

### Procedure for making the frozen stock of the transformants

A single colony was picked up and mashed in 10 μl of Aluotto liquid medium. The mashed colony-agar mixture was inoculated into 1 ml of Aluotto medium containing 15 μg/ml of gentamicin for 3–5 days in 24-well plates. Then, 1 ml of cultivated transformants were inoculated into 10 ml of fresh Aluotto medium containing 15 μg/ml of gentamicin and were cultivated for 3–4 days. The culture was divided into 500 μl aliquots and was centrifuged at 10,000 × *g*. The supernatant was removed completely, and the cell pellet was resuspended in 100 μl of PBS consisting of 75 mM sodium phosphate (pH 7.3), 68 mM NaCl, and 10 mM glucose. Further, the cells were centrifuged at 10,000 × *g* and resuspended in 500 μl of fresh Aluotto medium containing no antibiotics. The suspension was frozen immediately in liquid nitrogen and kept in a deep freezer.

### Fluorescence microscopy and protein analysis

Chemical fixation of the cells and immunofluorescence microscopy were performed as described previously^9,41,42^. TIRF imaging of the fixed samples was performed using an Olympus IX83 (Olympus, Tokyo, Japan) inverted optical microscope in TIRF mode. A 100 × TIRF objective (Plan-APO 100 ×/1.45 Oil, TIRFM, Olympus) was used to collect fluorescence onto an electron-multiplying charge-coupled device (EMCCD) camera (iXon X_3_, Andor, Oxford Instruments, UK) and excitation was provided by 514 and 532 nm lasers for EYFP and Alexa Fluor 555, respectively^16^. SDS-PAGE and western blotting analysis were performed as described previously^9^.

### Analyses of binding and gliding

Cultured cells were collected by centrifugation at 12,000 × *g* for 4 min at room temperature (RT) and then were washed and suspended in PBS/G. The cell suspension was inserted into a tunnel chamber with 5-mm width, 22-mm length, and 86-µm thickness. The tunnel chamber was constructed with a coverslip and a glass slide, assembled with double-sided tape and precoated with a growth medium containing 10% horse serum for 60 min. Mycoplasma gliding was observed by phase-contrast microscopy using a BX50 microscope (Olympus) and recorded with a Wat-120N CCD camera (Watec, Yamagata, Japan). All video data were analysed by using ImageJ software, version 1.52a (http://rsb.info.nih.gov/ij/), as previously described^27,31,33,37^.

### Sequence and phylogenetic analyses

Genome analyses and comparisons were carried out with MolliGen, a database for *Mollicutes* genomes (http://molligen.org). Sequence similarities were searched using PSI-BLAST (http://blast.ncbi.nlm.nih.gov/Blast.cgi) against the NCBI non-redundant protein sequence databank. The sequences of homologs were aligned by T-Coffee (http://www.igs.cnrs-mrs.fr/Tcoffee/tcoffee_cgi/index.cgi), and their phylogenetic trees were constructed by ClustalW (http://www.genome.jp/tools/clustalw/). The whole-genome sequences of *M. agassizii* and *M. testudineum* were provided by The Genome Portal of the Department of Energy Joint Genome Institute (http://genome.jgi.doe.gov). Protein sequences of *M. pulmonis* and *M. mobile* were named by their locus tags. PSIPRED v.3.0 (http://bioinf.cs.ucl.ac.uk/psipred/) was used to predict secondary structure. The TMpred (http://www.ch.embnet.org/software/TMPRED_form.html) and TMHMM (http://www.cbs.dtu.dk/services/TMHMM) methods were used in combination for the prediction of transmembrane segments. Species and protein phylogenies were determined by the neighbour-joining (NJ) and the maximum likelihood (ML). The reliability of each tree topology was checked by 1000 bootstrap replications using the MEGA7 package (http://www.megasoftware.net/). Operon constructions were predicted by DOOR^2^ (Database of prOkaryotic OpeRons, Version 2.0)^43^.

## Supplementary information

Supplementary information.

Supplementary information.
