# Supplementary material for: Identification and sequence analyses of the gliding machinery proteins from Mycoplasma mobile
Source: Sci Rep. 2020 Mar 2;10:3792. doi: 10.1038/s41598-020-60535-z (PMC7052211; doi:10.1038/s41598-020-60535-z)
Supplement: Supplementary file 1 — Supplementary information. [file 41598_2020_60535_MOESM1_ESM.pdf]

Table S1. Species names and NCBI accession numbers of focused genomes

**Species name**	**Abbreviation**	**Genome accession number**
Mycoplasma mobile 163K	MMOB	NC_006908.1
Mycoplasma pulmonis UAB CTIP	MYPU	NC_002771.1
Mycoplasma testudineum	MTES	PRJNA245595*
Mycoplasma agassizii	MAGAS	PRJNA245605*
Mycoplasma bovis PG45	MBOVPG45	NC_014760.1
Mycoplasma conjunctivae HRC/581T	MCJ	NC_012806.1
Mycoplasma fermentans JER	MFE	NC_014552.1
Mycoplasma arthritidis 158L3-1	MARTH	NC_011025.1
Mycoplasma agalactiae PG2	MAG	NC_009497.1
Mycoplasma hominis PG21	MHO	NC_013511.1
Mycoplasma hyopneumoniae 232	mhp	NC_006360.1
Mycoplasma hyorhinis HUB-1	MHR	NC_014448.1
Mycoplasma synoviae 53	MS53	NC_007294.1
Mycoplasma cynos C142	MCYN	NC_019949.1
Mycoplasma crocodyli MP145	MCRO	NC_014014.1
Mycoplasma anatis 1340	GIG	NZ_AFVJ00000000
Mycoplasma alligatoris	MALL	NZ_ADNC00000000.1

* NCBI BioProject accession numbers linked to "The genome portal of the Department of Energy Joint Genome Institute (JGI)".

	Nucleotide length in MMOB and orthologs (bp)			
**Gene annotation in *M. mobile***	**MMOB**	**MYPU**	**MAGAS**	**MTES**
1610	1443	1524	1554	1512
1620	882	939	918	957
1630	1011	1149	1002	1038
1640	597	558	555	588
1650	3477	2391+765	3285	3264
1660	1587	1590	1587	1584
1670	2355	2097	2247	2133
