# Supplementary material for: Identification and sequence analyses of the gliding machinery proteins from Mycoplasma mobile
Source: Sci Rep. 2020 Mar 2;10:3792. doi: 10.1038/s41598-020-60535-z (PMC7052211; doi:10.1038/s41598-020-60535-z)
Supplement: Supplementary file 2 — Supplementary information. [file 41598_2020_60535_MOESM2_ESM.pdf]

To Scientific Reports (e7555a1f-ce86-48e9-8016-46c4aaa44330) ver 2

Identification and sequence analyses of the gliding machinery proteins from *Mycoplasma mobile*

Isil Tulum, Kenta Kimura, and Makoto Miyata*

Department of Biology, Graduate School of Science, Osaka City University, Sumiyoshi-ku, Osaka 558-8585, Japan.

*Corresponding author: Makoto Miyata, miyata@sci.osaka-cu.ac.jp

Tel. +81 (6) 6605 3157; Fax +81 (6) 6605 3158.

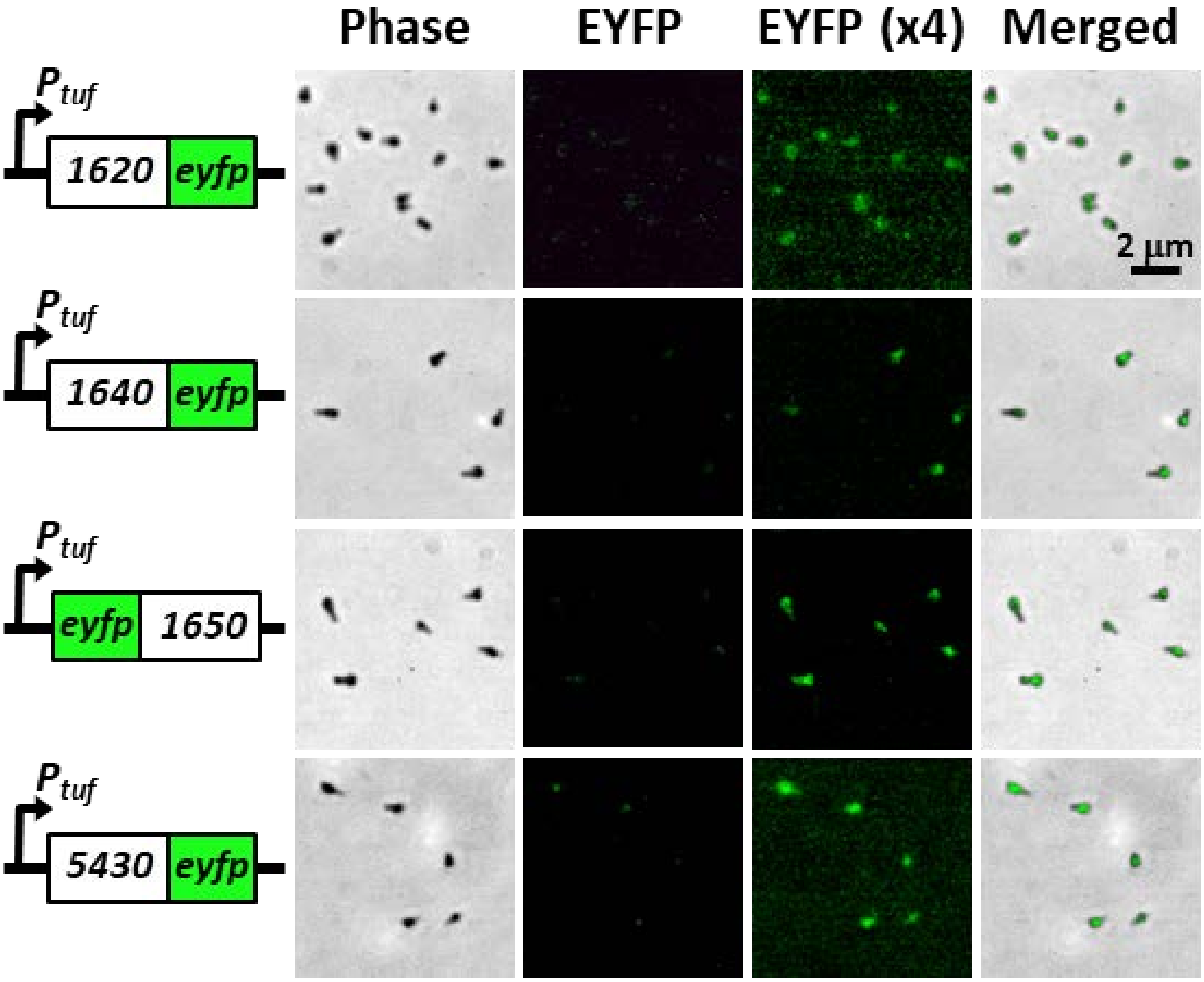

Figure S1. EYFP-fusion constructs showing low intensity signals. The C-terminal fusions of MMOBs 1620, 1640, 5430, and the N-terminal fusion of MMOB1650 are shown. Phase-contrast images are shown in the leftmost column. Fluorescence images obtained in conditions common with Fig. 3 and with four times longer exposure are shown in the second left and the second right panels, respectively. Merged images are shown in the rightmost column.

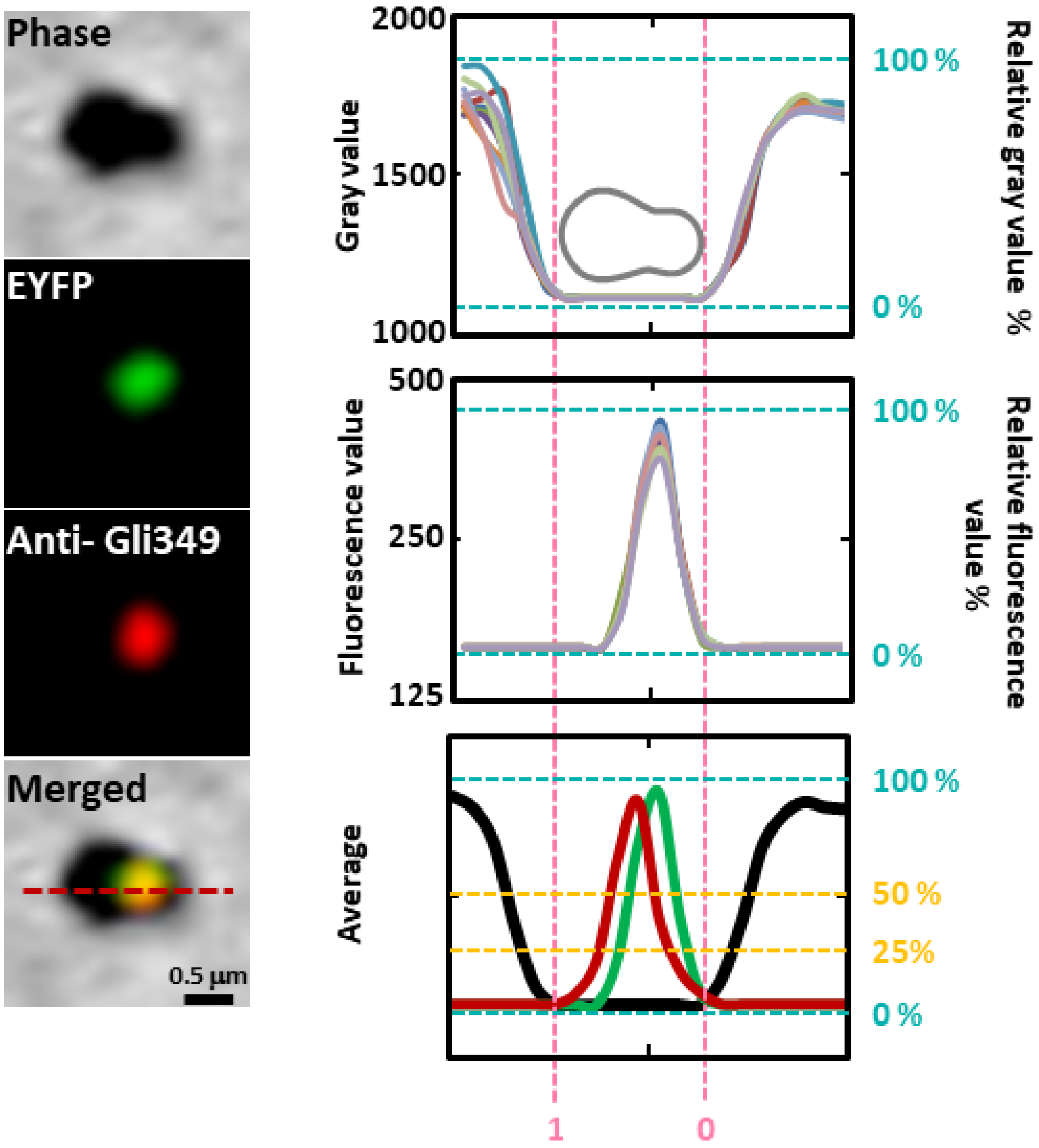

Figure S2. Schematic of the mapping procedure for the fluorescent foci. Images of phase-contrast, EYFP-MMOB1620, and anti-Gli349 antibody were analysed. They were profiled along with the red dashed line as shown in the merged image at the bottom left panel. The front and tail end positions of cell axis were determined from image density profile and normalised for all cells. The image intensities were averaged for 10 cells and integrated into a graph as shown in the upper two panels of the right column. The fluorescent signals were normalised by the intensities at the fluorescence peak and the glass surface as shown by blue broken lines. The positions of front and tail ends of a cell were estimated from the shoulder of the density profile in the phase-contrast image, as shown by the red dashed lines. The averaged profiles and cell positions were integrated into the bottom panel of the right column as coloured by black, green, and red, respectively for phase-contras density, EYFP signal, and Gli349 signal.
